# An Abrupt Mid-1970s Shift in UK Birth Seasonality and Its Implications for Chronobiological Studies

**DOI:** 10.1177/07487304251384348

**Published:** 2025-11-26

**Authors:** Timothy J. Hearn, David Whitmore

**Affiliations:** * Department of Genomic Medicine, University of Cambridge, Cambridge, UK; † Cell and Developmental Biology, University College London, London, UK; ‡ Australian Institute of Tropical Health and Medicine, James Cook University, Townsville, QLD, Australia

**Keywords:** seasonal birth rates, chronobiology, hormonal contraception, photoperiod correlation, reproductive trends

## Abstract

We present a comprehensive analysis of the historical fluctuations and rephasing of seasonal birth rates in the United Kingdom from 1955 to 2015. We analyzed monthly live-birth records for England and Wales together with national photoperiod and surface-temperature series to track the annual rhythm of human reproduction. Fast Fourier transforms confirmed a robust 12-month component across the entire record, but breakpoint tests located a sharp phase shift in 1974-1976. Before this transition, peak conceptions clustered tightly around the summer solstice and yielded a stable March birth maximum. After 1976, the rhythm decoupled: the spring peak in births collapsed, a secondary autumn peak emerged, and inter-annual phase variability more than doubled. Cross-correlation analyses showed that, up to 1974, photoperiod led birth counts by ≈11 months whereas temperature played only a minor role. Post 1976, photoperiod correlations disappeared and a weaker, inverse link with temperature persisted. Sliding-window statistics indicate that variability has narrowed again since the mid-1990s, hinting at partial re-stabilization of the seasonal pattern, now centered in late autumn conceptions. These results demonstrate that the mid-1970s marked a singular disruption of the United Kingdom’s reproductive calendar, coincident with the nationwide roll-out of freely available hormonal contraception and other social shifts. The findings urge caution when pooling pre- and post-1974 cohorts in genetic or epidemiological studies—such as those using UK Biobank—to explore season-of-birth effects. More broadly, they highlight the plasticity of human annual timing and the need to disentangle biological from socio-environmental drivers of reproduction.

Circannual rhythms, biological oscillations with a period close to 1 year, guide a range of physiological and behavioral processes in diverse species, including humans. These rhythms, believed to have evolved to synchronize organisms with predictable annual changes in their environment (Bronson, 2009), manifest in a variety of intriguing ways, one of which is reproduction (Sáenz de Miera et al, 2014).

Evidence for circannual rhythms governing reproductive behaviors and outcomes is abundant in mammals. Documented seasonal variations in birth rates span various species from rodents to ungulates and humans (Roenneberg and Aschoff, 1990a; Lincoln and Clarke, 1994). This reproductive rhythmicity is hypothesized to optimize birth timing to coincide with optimal environmental conditions, thereby promoting offspring survival (Karsch et al., 1984). In humans, seasonal reproductive rhythm is underwritten by the seasonality of successful conceptions (Roenneberg, 2004) and also includes changes in the human sex ratio, presumed to be due to periconceptional effects, such as micronutrient supply (Melnikov, 2015).

Photoperiod, the day-length relative to night, acts as a principal zeitgeber, or time-giver, for these annual rhythms (Goldman, 2001). Photoperiodic control of reproduction is pervasive in mammals, with substantial research conducted in seasonal breeders like sheep (Elliott, 1976; Beltran et al., 2022). Small mammals tend to use the increasing day-length of Spring to drive their reproductive response, as gestations periods are relatively short and the offspring can then take advantage of the food abundance that occurs at this time of year. Larger mammals, including many farm animals with longer gestation periods, tend to mate during the shortening daylengths of Autumn, such that offspring are born in the following Spring—again when food resources are abundant and weather conditions are improving. Photoperiod detection occurs via the retinohypothalamic tract, relaying this information to the pineal gland, resulting in melatonin production. This hormone modulates the reproductive axis, thus controlling reproductive event timing (Guh et al., 2019), including birth or conception timing. Amazingly in early sheep studies, the infusion of a melatonin profile in pinealectomized sheep could actually drive the expected response to different seasonal daylengths directly, revealing the key role of this hormone in these animals (Bittman et al., 1983).

In the case of humans living in the United Kingdom, the late 20th century brought societal and technological upheavals that might have affected these innate biological rhythms. A pivotal event was the introduction of hormonal contraception by the UK’s National Health Service (NHS; Cleland et al., 2012). While available from the 1960s (The National Archives, Kew, 1963), hormonal contraception became widely accessible in the United Kingdom only by the mid-1970s—a significant societal shift in reproductive control (Marks, 2010). In 1974, the contraceptive pill became freely available to all women, regardless of marital status, through the NHS, leading to a usage surge (Wellings and Kane, 1999). This followed the Family Planning Act of 1967 (UK, 1967), enabling local health authorities in England and Wales to provide contraceptive advice and supplies to anyone, irrespective of age or marital status, albeit with charges (Samuels, 1967).

The long history of birth records collected by the United Kingdom present an excellent opportunity to explore the potential interplay between hormonal contraception, circannual rhythms, and photoperiodic regulation of birth rates. The UK’s extensive, comprehensive historical birth records, maintained by the Office for National Statistics (ONS), started under the establishment of the registry office by the Births and Deaths Registration Act of 1836, mandating all births registered in England within 42 days (United Kingdom Parliament, 1836). Combined with public meteorological datasets, these records enable us to compare UK reproduction with environmental conditions (Roenneberg and Aschoff, 1990b) throughout most of the 20th century and examine long-term trends and historical shifts in birth rates (Roenneberg and Aschoff, 1990a).

In light of recent studies associating birth season with later health outcomes (Vaiserman, 2021) and the growing trend to correlate birth month or perinatal photoperiod with various traits using data from the UK Biobank, it becomes ever more crucial to understand birth rate patterns (Day et al., 2015; Escott-Price et al., 2019; Didikoglu et al, 2020; Lewis et al., 2021, 2024; Viejo-Romero et al, 2024). Reports have suggested associations between chronotype and birth season (Mongrain et al., 2006; Huang et al., 2015), hinting at the fascinating coupling of circa-annual and circadian rhythms. However, the pronounced shift seen in seasonal time of European births (Roenneberg, 2004) raises questions about the legitimacy of such correlations, when analyzing cohorts of participants born across this time period, if this shift is also seen in UK data.

This study sought to ascertain whether the trend observed in Germany (Lerchl et al., 1993), Poland (Nenko et al., 2022), and Spain (Cancho-Candela et al., 2007), overt seasonality, with a pronounced shift in the late 20th century during the 1960s and 1970s (Roenneberg, 2004), is reflected in UK data, and if so, when this deviation from robust seasonal birth rhythms occurred. We then compare the early- (1955-1974) and late-twentieth-century (1976-2014) periods to see whether photoperiod or temperature better tracks the annual rhythm in births.

## Methods

### Data Accessibility and Computational Environment

All data used in this study are publicly available from the UK government. Data on live births were obtained from the Office for National Statistics (ONS) under the *Birth Characteristics in England and Wales* dataset (Live births - Office for National Statistics, n.d.).

Environmental data, including gridded monthly values of hours of sunshine and air temperature, were sourced from the UK Met Office through *data.gov.uk*. These datasets, known as UKCP09 gridded land observations, were retrieved in 10-year blocks covering the period from 1929 to 2014 (CEDA Archive Web Browser, n.d.). To represent conditions in England and Wales, the average of the gridded Easting-Northing values was used.

Together, these datasets provide complete coverage of monthly birth rates and environmental conditions from 1953 to 2014. Based on visual inspection of the time series, 2 distinct periods were identified: 1953-1974 and 1976-2014. Separate datasets were prepared for analysis, each containing records of birth rates (BR), light levels, and temperature readings.

All computations were carried out in Python 3.11 using *numpy* 1.26, *pandas* 2.2, *statsmodels* 0.15, and *matplotlib* 3.9; the accompanying code reproduces every step described here.

### Code Availability

The full dataset and analysis code are available in the GitHub repository: https://github.com/comparativechrono/Rephasing-of-Seasonal-Birth-Rates-in-the-United-Kingdom-/.

### Data Preparation and Gestation-Based Conception Estimates

Monthly birth counts from January 1955 through December 2014 were read into pandas and combined with calendar information. To infer the month of conception, we shifted each birth datum back by 9 calendar months (≈280 days) on the DateTime index.

### Dual-Axis Plot

We used Matplotlib to create a primary y-axis for raw birth counts and a twin secondary y-axis for the month-of-year index of peak conceptions. The secondary axis was scaled from 6 (June) to 13 (January) to encompass exactly the 8 distinct months during which conception maxima occurred; months 2-5 (Feb-May) never emerged as modal conception months and were omitted to keep focus on the active band.

### Seasonal Comparison and Percentage-Difference Bar Plot

To compare the monthly birth-rate profile before (1955-1974) and after (1975-2014) the mid-century shift, we grouped the data by calendar month and computed the mean births per month for each epoch. The percentage difference for each month m was then calculated as:

$$\Delta_{m}=100\times \frac{{\bar{B}}_{m}^{\left(\;1975–2014\right)}-{\bar{B}}_{m}^{\left(\;1955–1974\right)}}{B_{m}^{-\;\;(\;1955–1974)}}\;.$$

### Bootstrapped Uncertainty Estimation

To quantify sampling variability in $\Delta_{m}$ , we performed 1000 bootstrap replicates: for each replicate b , we drew with replacement 12 monthly-mean birth values from the 1955-1974 group and 12 from the 1975-2014 group, computed the corresponding percentage difference $\Delta_{m}^{\left(b\right)}$ , and then recorded the distribution of ${\left\{\Delta_{m}^{\left(b\right)}\right\}}_{b=1}^{1000}$ . The standard error for each month’s difference was taken as the standard deviation of its bootstrap distribution:

$${\mathrm{SE}}_{m}=\sqrt{\frac{1}{999}\overset{1000}{\underset{b=1}{\sum }}{\left(\Delta_{m}^{\left(b\right)}-\overset{-}{\Delta_{m}}\right)}^{2}}\;.$$

These ${\mathrm{SE}}_{m}$ values were plotted as error bars on the bar chart of $\Delta_{m}$ providing a “± 1 SEM” uncertainty interval around each monthly percentage change. All plotting was done in Matplotlib, data manipulation in pandas/NumPy, and bootstrap sampling via NumPy’s random choice.

### Visualization of Seasonal Birth Patterns (Birthogram)

For each calendar year the 12 monthly counts were rescaled to a 0-1 interval (min-max normalization) so that inter-year comparisons reflected the shape of the annual cycle rather than absolute magnitude. The normalized sequence for year *n* (January-December) was concatenated with the independently normalized sequence for year *n* + 1, yielding a 24-month vector that preserves continuity across the December-January boundary. These vectors were stacked vertically in chronological order and plotted as filled bands (actogram format), with the earliest year at the top. The inferred peak conception value recorded for each year was placed as a marker at its corresponding month position, allowing visual tracking of the peak’s temporal drift.

### Spectral and Phase Analysis

To characterize the periodic structure of UK birth rates and quantify shifts in annual peak timing, we conducted 2 complementary analyses: (1) fast Fourier transform (FFT)–based spectral decomposition, and (2) year-by-year phase estimation via cosine-model fitting and circular statistics.

Monthly birth counts from January 1955 to December 2014 were first split into 2 epochs (1955-1974 and 1976-2014) to capture pre- and post-phase-shift behavior. Within each epoch, the raw counts were mean-centered and scaled to unit variance (*z*-scored) so that the FFT amplitudes would be directly comparable across unequal sample sizes. We then applied NumPy’s FFT routine to each *z*-scored series. The corresponding frequency bins were inverted to obtain periods in months, and only those frequencies corresponding to periods between 1 and 20 months were retained. Spectral amplitudes were normalized by dividing the absolute Fourier coefficients by the number of observations (N) in each epoch, yielding dimensionless spectral amplitudes.

To isolate the annual component for each calendar year, we removed long-term trends from the full 1955-2014 series by subtracting a 13-month centered rolling mean. For each year, the resulting residuals (months 1-12) were fit to a 4-parameter cosine model.

$$y\left(x\right)=\mathrm{Acos}\left(\frac{2\pi x}{12}+\phi \right)+C,$$

using SciPy’s curve_fit with bounds enforcing a 12 ± 1 month period and phase ϕ∈[−π,π] . The optimized phase parameter ϕ (in radians) represents the timing of the annual phase relative to January 1.

Yearly phase estimates were plotted on a polar axis, with the angular coordinate (ϕ) denoting the timing of the cosine-model peak (0 rad = January) and the radial axis representing the calendar year. Successive points are connected to highlight gradual versus abrupt shifts. We computed the circular mean, circular standard deviation, and approximate 95 % confidence intervals of the ϕ distributions separately for pre-1975 and post-1975, using vector-sum methods ( $\bar{\phi }=arg(\sum e^{i\phi_{j}})$ ) and the standard error $\sqrt{-2\ln R}/\sqrt{n}$ , where *R* is the mean resultant length. These statistics provide distribution-based confirmation of both the delayed mean phase and increased inter-annual variability after the mid-1970s shift.

### Sliding-Window Bootstrap and Statistical Comparison

To assess how inter-annual variability in the timing of the birth-rate peak evolved over the study period—and to determine which post-1974 intervals differ from the early twentieth-century baseline—we applied a 10-year sliding-window analysis coupled with a nonparametric bootstrap test. First, for each window (advancing in 1-year steps through 1955-2014), we computed the circular standard deviation of the yearly peak-phase angles $\left\{\phi_{j}\right\}$ , using

$$\sigma =\sqrt{-2\;\ln|\frac{1}{n}\overset{n}{\underset{j=1}{\sum }}e^{\;i\phi_{j}}|\;}\;.$$

Within the same window, we generated 1000 bootstrap resamples (sampling with replacement) of its n phase angles and recalculated σ for each resample to derive a 95% confidence interval (2.5th-97.5th percentiles) around the observed σ.

Next, to test whether variability in each post-1974 window differed from the baseline period (1955-1974), we constructed a separate bootstrap distribution of σ from 1000 resamples of the entire baseline phase series. For each sliding window’s observed σ , we computed a 2-sided *p*-value as

$$p=2\times \min\{\;\mathrm{Pr}(\sigma_{\mathrm{base}}\geq \sigma_{\mathrm{obs}}),\;\mathrm{Pr}(\sigma_{\mathrm{base}}\leq \sigma_{\mathrm{obs}})\},$$

where $\sigma_{\mathrm{base}}$ denotes a bootstrap draw from the baseline. Windows whose midpoint year lay after 1974 and for which *p* < 0.05 were flagged as exhibiting a statistically significant change in phase variability relative to the early baseline. Significant windows are marked with an asterisk in the sliding-window plot.

### Seasonal-Component Removal

To isolate seasonal variability, each series was decomposed using seasonal-trend decomposition based on LOESS (STL) implemented using statsmodels.tsa.seasonal.STL (see supplemental methods). The estimated seasonal component was subtracted from each original series, yielding seasonally adjusted residuals that retain both trend and irregular fluctuations.

### Rolling-Window Cross-Correlation

To characterize the evolving association between each environmental driver $x_{t}$ (light, photoperiod, or temperature) and the biological response $y_{t}$ (birth rate), we applied a sliding-window cross-correlation analysis. A window of N=60 consecutive months was advanced 1 month at a time along the record; for every such window w beginning at calendar index t and for every integer lag ℓ from −20 to 0 we formed 2 length − N−|ℓ| subsequences and calculated their Pearson correlation. When ℓ≥0 the environmental series was shifted forward so that the pair $\left(x_{t+\ell +k}^{\left(w\right)},y_{t+k}^{\left(w\right)}\right)$ was evaluated for k=0,…,N−ℓ−1 , meaning the driver is allowed to *lag* the response by up to 20 months. Conversely, when ℓ<0 the biological series was shifted forward and the pair $\left(x_{t+k}^{\left(w\right)},y_{t-\ell +k}^{\left(w\right)}\right)$ was evaluated for k=0,…,N+ℓ−1 ; here the driver leads the response. These 2 cases can be written compactly as

$${\hat{\rho }}_{xy}^{\left(w\right)}(\ell )=\{\begin{array}{cc} \mathrm{corr}(x_{t+\ell }^{\left(w\right)},\;y_{t}^{\left(w\right)}), & \ell \geq 0,\\ \mathrm{corr}(x_{t}^{\left(w\right)},\;y_{t-\ell }^{\left(w\right)}), & \ell <0, \end{array}$$

where corr is the unbiased Pearson estimator applied to the aligned samples. Repeating this calculation for every lag and every window yields a matrix $R_{xy}$ whose rows correspond to lags and whose columns step forward in calendar time; an analogous matrix $R_{xy}^{*}$ is obtained from seasonally adjusted residuals to isolate inter-annual covariation.

## Results

**Figure 1.**
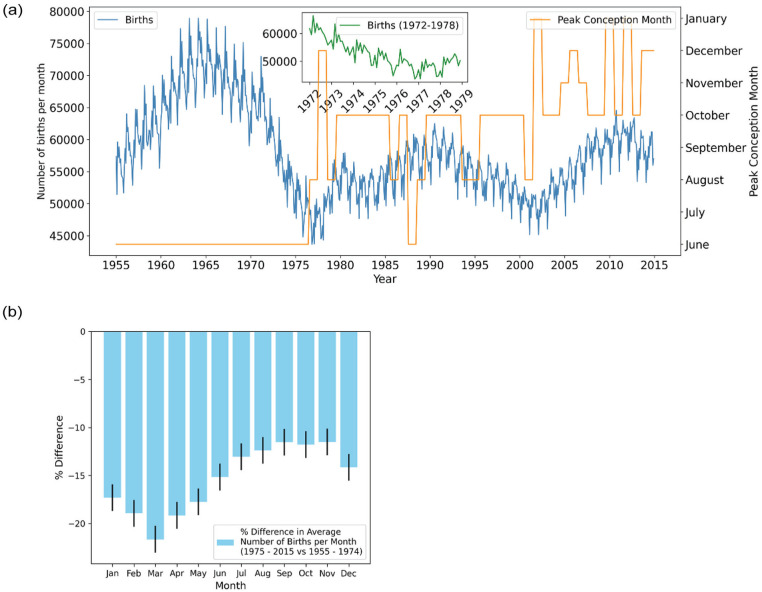
Shift in the phase of the annual rhythm of UK birth rates in the mid-1970s. (a) The figure presents an overlay of 2 datasets from 1955 to 2015: the number of births per month (blue line) and the inferred peak conception month (orange line). The peak conception month represents the estimated month with the highest number of conceptions leading to live births. Months outside this June-January window were never modal and are therefore not shown. An inset plot (green line) zooms in on births between 1972 and 1979, highlighting the period where the shift in peak conception occurred. (b) A bar plot illustrating the percentage difference in the average number of births per month between 2 periods: 1955-1974 and 1975-2015. Each bar represents the percentage change for a specific month, with bar height indicating the magnitude of the difference. Error bars denote the standard error, calculated using a bootstrapping method (1000 samples), providing a measure of uncertainty in the estimates.

The UK birth data from 1955 to 2015 reveals an annual pattern with cyclic behavior (Figure 1a). At the beginning of the reporting period a pronounced peak in births is observed in the third month of the year, followed by a gradual decline over the year and a sharp reappearance of the peak the following March. Prior to 1953, the UK records had quarterly birth data collection; however, the maximum birth rate observed in the first quarter had been consistent since 1838 (records start from September 1837) and persisted for over 150 years, indicating a clear biological pattern (Supplemental data 1). In the more detailed monthly data, this pattern remains evident until well into the 20th century, with the characteristic spring peak still observable in Figure 1a up until 1974.

**Figure 2.**
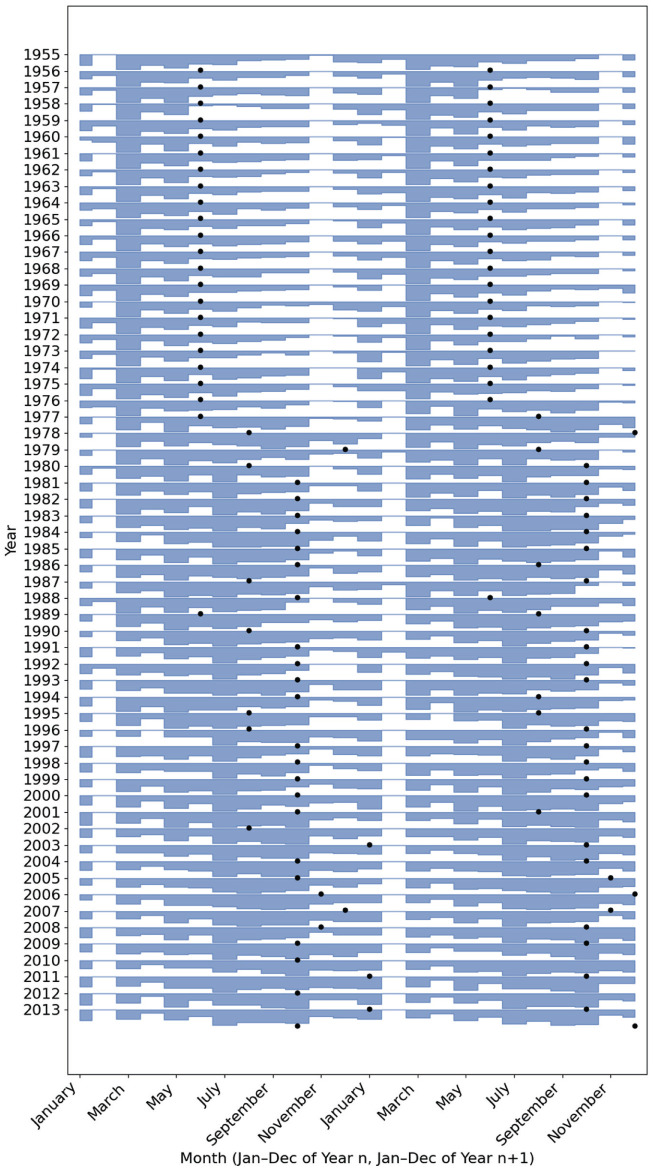
Double-plotted “birthogram” of monthly live births, 1955-2014. Each horizontal band displays 2 consecutive calendar years: January-December of year *n* (left half, x-axis) followed by January-December of year *n* *+* 1 (right half, x-axis). For every 12-month segment, UK birth counts were min-max normalized to the 0-1 range and plotted as a filled area; rows are vertically offset so the earliest year appears at the top. Black dots mark the inferred peak-conception month for each year.

From 1974 to 1978, the annual birth rate’s shape changes significantly, with the single spring peak flattening and a second, smaller crest appearing in early autumn, giving the seasonal profile a broader, double-peaked shape. This change is clearly illustrated in the inset figure of Figure 1, with the winter arm of the bimodal curve predominant by 1978, with only a small Spring peak. To visualize the transition more intuitively we generated a “birthogram” (Figure 2)—a double-plotted actogram in which each row displays January-to-December of year *n* followed by January-to-December of year *n* *+* 1. In this display, the spring peak is seen to weaken progressively after 1974, while a second peak within the autumn months strengthens. By the late 1970s, the autumn peak overtakes the spring peak; thereafter the dominant peak remains anchored in the Autumn, getting progressively later in subsequent decades, with only a minor peak in March.

To analyze the data through a biological lens, we can assume that conception occurs 9 months prior to birth. Although about 7% of live births at the end of the study period are pre-term (ONS, 2016), which can impact time-series analysis (Darrow et al., 2009), this assumption enables us to approximate peak conception prior to 1975 around the summer solstice (Figures 1a and 2), as previously demonstrated by Roenneberg and Aschoff (1990b), hinting at an association with photoperiod.

To summarize—before 1974, the birth peak consistently occurs in March, while after 1976, the peak gradually shifts later into the year, starting with a bimodal response, with 1975 as a breakpoint. This might suggest that the seasonal slump in conception associated with winter has been lost, while the spring birth rate remains at the basal rate. Interestingly, a roughly 25-year cycle in birth rates also appears to be present, with peaks roughly in epochs centered on 1965, 1990, and 2015.

Because the raw curve and the overall decline in births makes it difficult to see which part of the annual cycle shrank most, we averaged births for 1955-1974 and for 1975-2015 and plotted the percentage change for each calendar month (Figure 1b) with an estimate of the standard error of the percentage difference using bootstrapping. Although births fell in every month, the reduction was far from uniform: the largest change occurs in March, with over a 20% reduction in births, whereas autumn months declined by only 11%-12%. To further investigate this, we looked at the overall drop in amplitude of the periodic seasonal component by extracting the seasonal component of the birth rate using seasonal trend decomposition by Loess (STL, Supplemental Figure 1). This revealed that amplitude actually starts to collapse prior to the 1975 breakpoint, where it reaches a plateau, and decreases from 1968 onward, therefore perhaps reflecting the overall drop in birth rate seen in the latter half of the 20th century. Thus, in a backdrop of declining amplitude of seasonal births, we observe the loss of the strong peak of births in the Spring and the smallest decrease in Autumn, which due to the overall reduction in births initially appears as a bimodal curve.

**Figure 3.**
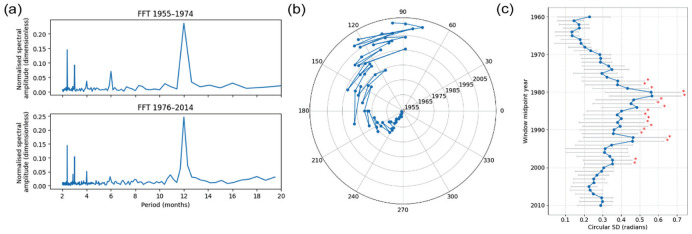
Comparative fast Fourier transform (FFT) analysis of UK birth rate data for, 1955-1974 and 1976-2014 and annual variation in phase over time. (a) Fast Fourier transform (FFT) spectra of *z*-scored monthly birth counts convert the data from the time to the frequency domain, revealing periodic components. The x-axis represents the periods (in months) and the y-axis shows normalized spectral amplitude (dimensionless) obtained from *z*-scored monthly birth counts. Periods considered range between 1 and 20 months. The upper plot (FFT 1955-1974) presents FFT analysis for the birth rates from 1955 to 1974, whereas the lower plot (FFT 1976-2014) illustrates the FFT analysis for the birth rates from 1976 to 2014. In both eras the dominant peak occurs at 12 months, confirming the persistence of an annual rhythm, with a smaller harmonic at 6 months, even though its phase later shifted. (b) Polar plot of annual birth-rate peak phases, obtained by fitting a cosine model to each year’s detrended monthly birth counts. The angular coordinate (ϕ) indicates timing of the peak relative to January (0°= January, with 30° increments for each subsequent month). The radial coordinate corresponds to calendar year, with 1955 at the center and 2014 at the outer edge (radial grid lines labeled every 10 years). Blue dots mark the phase for each year, and a continuous line connects successive years to allow easy identification of the gradual drift of the seasonal peak over time. (c) A forest plot showing 10-year sliding-window estimates of inter-annual variability in birth-rate peak timing. Each blue circle marks the circular standard deviation (σ, in radians) of the annual peak-phase angles computed for a window centered on the corresponding year. Horizontal gray bars show the 95% confidence intervals obtained from 1000 bootstrap resamples of each window’s phase angles. Red asterisks above select points denote windows (mid-point years) after 1974 whose σ differs significantly (2-sided *p* < 0.05) from the 1955-1974 baseline distribution.

Following the methodology used in previous studies for consistency, we analyzed the annual rhythm with frequency analysis tools to verify that a robust annual component persisted throughout the study period empirically (Figure 3). We applied a fast Fourier transform (FFT) to *z*-scored monthly birth counts. Both intervals showed a dominant 12-month spectral peak, with a smaller 6-month harmonic, although surprisingly the post-1976 series exhibited a drop in the 6-month peak—indicating that the annual rhythm persisted after 1974 (Figure 3a). We then applied a yearly cosine-fit to the detrended birth-rate residuals (13-month rolling mean subtraction) in order to extract, for each calendar year, the phase of the annual peak in radians (Figure 3b). This clearly shows a progressive delay of the seasonal birth-rate maximum across the mid-1970s phase shift, as well as a noticeable increase in inter-annual variability afterward.

To quantify the shift in timing and the change in variability of the annual birth-rate peaks, we used circular statistics on the year-by-year phase angles. For the pre-1975 period, the circular mean phase was –2.29 radians (95 % CI: –2.41 to –2.16) with a circular standard deviation of 0.29 rad, indicating a tightly clustered annual peak. In contrast, in the post-1975 era the mean phase shifted to 2.32 rad (95 % CI: 2.12 to 2.51) and the circular SD increased to 0.64 rad, reflecting both a later average timing and greater inter-annual dispersion. These results provide robust, distribution-based evidence that the birth-rate rhythm not only delayed by approximately 4.6 months (2.32 rad from a January zero) after the mid-1970s but also became significantly more variable from year to year, agreeing with the peak analysis in Figure 1, thus demonstrating that the change in peak time is consistent with a change in phase analysis using conventional temporal rhythm analysis tools. Qualitatively, it appeared that the birth-rate peak has become less variable in recent years. Restricting our analysis to the most recent 20 years (1995-2015) shows that the annual birth-rate peak has become markedly more stable: the phase estimates span only a 0.54 radian range, with a circular mean of 1.88 rad (95 % CI: 1.74-2.01 rad) and a circular standard deviation of 0.31 rad—essentially identical to the pre-1975 variability (SD = 0.29 rad). To chart how this variability evolved, we then applied a 10-year sliding-window bootstrap procedure, computing the circular SD (with 95% bootstrap CIs) for each window and testing it against the 1955-1974 baseline, generating a forest plot (Figure 3c). Variability remained low (≈0.15-0.25 rad) through the 1950s and 1960s, climbed steeply in the late 1970s—peaking around the 1980 window midpoint at ≈0.55 rad—and thereafter declined. SDs were consistently significantly elevated relative to baseline (*p* < 0.05) from this period until 1993. Together, these results suggest that, after a period of increased dispersion following the mid-1970s phase shift, the seasonal rhythm in UK birth timing has begun to re-stabilize over the past 2 decades.

We have seen that the seasonal variation in birth rates showed a marked discontinuity in the mid-1970s. This singular step change, rather than a gradual drift, motivated a formal search for a structural break in the relationship between birth seasonality and candidate seasonal environmental correlates. Data for the average UK monthly light exposure and temperature from the Office for National Statistics (ONS) is available from 1953 to 2014, enabling us to identify trends between these monthly datasets.

To detect the break quantitatively we fitted an ordinary least squares regression of the monthly birth seasonal component on the contemporaneous seasonal components of light, photoperiod and temperature and applied the Chow test at every candidate month between January 1965 and December 1985 (Supplemental Figure 2). The resulting *F* statistic rose sharply to a plateau that peaks in May 1976 (*F* ≈ 81, *p* < 0.001). Any breakpoint between mid-1975 and early 1976 yields essentially the same gain in fit (Supplemental Table 2), confirming a single regime shift centered on the mid-1970s.

**Figure 4.**
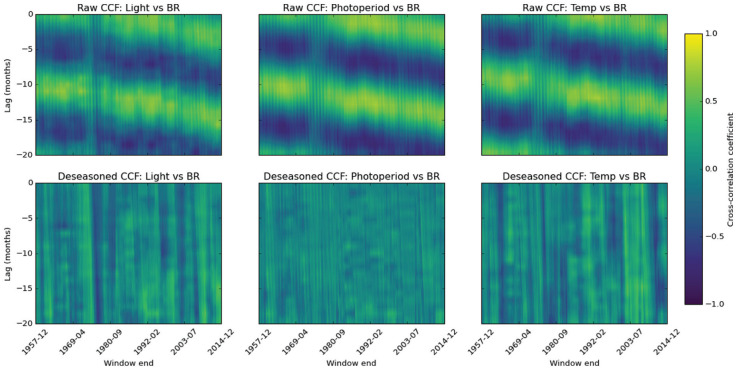
Sliding-window cross-correlation of potential environmental associations with birth rate (BR) show that following deseasonalization correlations vanish. Coherent horizontal bands reveal persistent lead-lag relationships, whereas vertical structure highlights periods when a driver-response linkage strengthens or weakens through time. Each panel displays Pearson correlation coefficients calculated in 60-month windows that advance 1 month at a time across the study period. Negative values indicate that the driver leads birth rate. Columns correspond to the 3 drivers—incident light, astronomical photoperiod, and surface temperature. The upper row shows correlations computed from the raw series (with the mid 1970s disruption clearly visible as weakened linkage) and the lower row shows correlations after removal of the deterministic seasonal component by robust STL decomposition. Cell color encodes coefficients on a symmetric scale from −1 to 1 (shared color bar at right), where warmer hues denote positive associations and cooler hues negative associations.

The nature of the instability was explored with rolling 60-month cross-correlation functions (CCFs) between births and each environmental variable over lags −20 to 0 months. Before the mid-1970s in raw cross-correlation function maps, all 3 variables show strong, seasonally driven peaks at the expected multiples of 12 months, with banded regions of high positive correlation consistent with the shared annual cycle underlying their relationship with birth rate (Figure 4). However, this regular pattern breaks down in the mid-1970s, revealing a loss of the usual seasonal synchrony. Following this, the pairwise correlations begin to drift, consistent with the phase change in birth rate that we have observed. When CCF is applied across both epochs (Supplemental Figure 3), rather than a rolling fashion, for 1953-1974, the strongest correlation is found with photoperiod at 11 months prior to peak birth rate (*r* = 0.337), and a weaker association (*r* = 0.292) is found with temperature with a 9-month lag to peak birth rate. Conversely, for 1976-2014, the association with light duration weakens (*r* = 0.337).

To determine whether the disruption reflects genuine changes in lead-lag dynamics or simply altered seasonality, we applied STL decomposition to remove each series 12-month component. By stripping out the dominant oscillation, the deseasoned CCF maps (Figure 4) isolate any residual lead-lag effects: if correlations were purely seasonal, they would collapse to noise once the cycle is removed, whereas persistent peaks would signal non-seasonal lead-lag associations. Indeed, after seasonal adjustment, all but episodic correlations largely vanish.

After removing the annual seasonal component from births, light, and temperature with STL, we also computed cross-correlations of the resulting anomalies over conception-to-birth lags (−20 to −0 months) for the 2 Chow-defined epochs, 1953-1974 and 1976-2014 (Supplemental Figure 3). With |*r*| > 2/√N (± 0.092) as the 95% significance threshold, light anomalies never reached significance in either period (peak |*r*| = 0.089 in the early epoch, 0.092 in the late epoch). Temperature anomalies, by contrast, were significantly negatively correlated with birth anomalies throughout, but the association weakened: the strongest coefficient declined from *r* = −0.203 at a 13-month lead before 1975 to *r* = −0.122 at a 9-month lead after 1975. Thus, while photoperiod-related variables were no longer significantly associated the timing of births, temperature retained a modest association, albeit with reduced effect.

Because epoch-wide averages can mask brief phase shifts, we extracted the strongest birth-environment lag in every 60-month window—each window being labeled by its largest CCF, however small—and regressed that monthly peak-lag series on calendar year within the 2 Chow-defined epochs (Supplementary Table 3) to pinpoint when any statistical coupling emerged, drifted, or disappeared over time, due to the shifting birth phase. In 1955-1974, as expected, the raw regressions show little movement apart from a modest advance relative to light (−0.08 month yr−¹, *p* = 0.03). Post-break (1976-2014) raw lags for all cues drift later (+0.08 to +0.19 month yr−¹, all *p* < 10−4), again as expected, but deseasoned regressions reveal light cues now advancing (~−0.06 month yr−¹, *p* ≈ 0.02), meaning the point of maximum birth-photoperiod statistical coupling is occurring earlier each year.

Altogether, the results are consistent with a single narrative: UK birth seasonality experienced an abrupt statistical decoupling from annual variables in the mid-1970s, after which light and day-length associations became fleeting and statistically unstable, while temperature continued to show a modest but persistent association. The Chow test locates the structural break squarely in 1975-1976; rolling CCF maps show a collapse of the regular 12-month correlation bands at that point; and seasonally adjusted, epoch-wide calculations confirm that only the temperature-birth link remains consistently significant thereafter.

## Discussion

The persistent seasonal oscillation in human reproduction rates in the United Kingdom, reflecting photoperiodic changes prior to 1953, is compelling. Despite socio-economic impacts causing birth rate fluctuations, a pronounced annual rhythm was consistently observed, with a stable peak of conceptions around the summer solstice in June. This photoperiodic regulation of birth rates (Cummings, 2010) aligns with early 20th-century biological trends varying with latitude (Moos and Randall, 1995), corroborating existing discussions on human seasonal photoperiodicity (Bronson, 2004; Roenneberg, 2004).

Efforts to decipher the seasonality of human conception have given rise to numerous theories, such as age-related seasonal fertility (Rizzi and Dalla-Zuanna, 2007). Seasonal influences on successful embryo implantation and IVF outcomes have generated diverse findings. While some studies report no effects (Dunphy et al., 1995; Wunder et al., 2005), others document greater oocyte yield and higher fertilization rates during extended photoperiods (Rojansky et al., 2000; Wood et al., 2006). Conversely, several studies find no significant difference (Revelli et al., 2005; Xiao et al., 2018; Liu et al., 2019).

Despite the inconsistency in seasonal impact on embryo implantation, there is consensus regarding the annual variation in male contributions to seasonal reproduction. Evidently, human sperm production fluctuates seasonally (Levine, 1994). Changes in sperm motility and morphology (Ozelci et al., 2016), alongside sperm chromatic condensation variations (Henkel et al., 2001), partially elucidate the seasonal variation in human reproduction (Rojansky et al., 1992). Photoperiod and latitude may provide historical seasonal birth rate insights; however, the contemporary birth rate’s seasonal responses are more challenging to predict due to myriad influences (Lam and Miron, 1991; Boland et al., 2020).

We show that the United Kingdom’s long-standing March birth peak collapsed abruptly between 1974 and 1976, shifting ~4 months later and remaining unstable for 4 decades. This had been observed in partial data previously (James, 1990), and we confirm here using multiple change-point and spectral tests which converge on this single, structural break, raising the question of potential triggers. Hypothetical factors affecting birth patterns in the 20th century could include environmental changes or technological advancements, as well as social-economic changes (Bobak and Gjonca, 2001; Dahlberg and Andersson, 2018). For instance, electric light, introduced to Britain in the 1930s, might have disrupted night-time darkness, essential for many photoperiodic organisms, though this is less likely to have impacted human behavior. The proliferation of central heating systems predominantly in the 1970s and 1980s could also have played a role, as heating has a known impact on reproduction (Barreca et al., 2018). The significant, albeit very small, negative association with temperature we find post 1976 lends some support to this hypothesis.

However, the drop in births in the mid-1970s has previously been noted on an annual scale, and this drop has been linked to oral contraceptive use (Wellings and Kane, 1999). In 1974 the National Health Service Reorganisation Act (1973) came into force (United Kingdom Parliament, 1973). This established family planning service by amending the first section of the NHS act of 1952 covering charges for drugs, medicines and appliances to now include contraceptive substances and appliances, and repealing the 1967 family planning act which had allowed such charges for contraceptive substances as the health authority saw fit (United Kingdom Parliament, 1952, 1967). This meant that oral contraceptives were made available for free on the NHS for unmarried teenagers and women for the first time, due to the exemptions in the 1952 act (United Kingdom Parliament, 1952). That this correlates with the change in phase of the annual birth rhythm in the United Kingdom as shown here, raises the possibility of the use of oral contraceptive affecting the seasonal reproductive rhythm. To support this hypothesis, we can look at the previously published collapse of seasonal birth rates in other European countries, and see that they also align with the introduction of oral contraceptives, such as Spain in 1964 (Marks, 2010). Therefore, the staggered national rollouts of free contraception in countries with high-quality birth registries could be viewed as a sequence of natural experiments.

Any correlation is obviously not clear cut, however, as concurrent with oral contraceptive use, societal changes emerged, including increased women’s liberation movements, greater female workforce participation, and family planning practice changes (Goldin, 2006). Legislation such as the 1970 Equal Pay Act may have affected childbirth timing as women sought to balance career and family (United Kingdom Parliament, 1970). The late 1970s also witnessed other reproductive technology advancements, including in vitro fertilization development (Steptoe and Edwards, 1978), which currently contributes about 3% of UK live births (HFEA, 2025). Such advancements may have further disrupted the circannual rhythm by uncoupling reproduction from natural physiological cycles.

The recent return of a more stable annual birth rate peak could suggest a partial recovery of the annual rhythm, or the establishment of an entirely new rhythm. That any rhythm, likely influenced by seasonal changes to sex hormone dial oscillation, persisted, albeit with a shifted phase, would require the existence of multiple oscillatory elements within the network. These elements, either social or biological, could be associated with shifts in the mother, embryo, or the United Kingdom’s genetic landscape. We have shown that the rhythm exhibits greater variability in peak time compared to the pre-contraception era, potentially reflecting changes in contraceptive use, societal attitudes toward family planning. For example, some couples may cease contraceptive use during certain seasons, inaccurately expecting immediate conception (Rodgers and Udry, 1988). The rhythm in this period is more closely associated with non-seasonal temperature than photoperiod, which are a known environmental influence on human fertility (Lam and Miron, 1996). This indicates the possibility of a circannual network surrounding reproduction in humans. Birth season can influence a range of characteristics, such as height (Douros et al., 2019; Candela-Martínez et al., 2022), coeliac disease (Daniel et al., 2019), childhood and gestational diabetes (Samuelsson and Ludvigsson, 2001; Khoshhali et al., 2021), autism (Lee et al., 2019), personality (Lee et al., 2021), and even the seasonal sex ratio correlating with peak conception (Kovanen et al., 2010; Melnikov, 2015).

This study was motivated by an increase in season of birth genetics research, particularly within chronobiology, enabled by large scale genomic resources such as the UK Biobank. There is intriguing evidence of the circadian clock influencing seasonal reproduction through associations between *ARNTL* and *NPAS2* clock variants (Kovanen et al., 2010) and seasonal reproduction variation. In addition, birth month association with chronotype predisposition (Jensen et al., 2021) suggests the intriguing possibility of a transgenerational circadian influence on reproduction. Individuals born in the summer exhibit *NPAS2* hypermethylation (Didikoglu et al., 2022), and reports suggest sex differences in birth-associated chronotype (Huang et al., 2015). Given the appealing prospect of human reproduction demonstrating strong seasonality, and the potential for chronobiology research that this gives, it is essential to underscore the seasonal response change in this fundamental biological property during the latter half of the 20th century. Any study that pools pre- and post-1974 UK births, or uses birth-month as a proxy for perinatal light exposure, blends 2 biologically distinct regimes and risks attenuating or reversing associations. We therefore advise caution when utilizing pooled pre-1974 and post-1976 data for UK chronobiological studies or attempting to establish associations between birth season and post-1976 life traits, as effect-size estimates that treat summer versus winter births as uniform across the 20th century are likely biased. The arrival of large scale UK genomic datasets such as the UK biobank has seen such epidemiological studies begin to be attempted (Didikoglu, Canal et al., 2020). Despite its potential, the UK Biobank may not provide entirely reliable photoperiodic human responses, considering most participants were born following this transition and future work should ideally stratify by the birth epochs we have identified here.

Our study is restricted to aggregated monthly counts; therefore, individual level confounders such as maternal age and migration could not be adjusted. Mechanistic inference is therefore indirect and while correlations in this study provide insights into UK human reproduction dynamics, these relationships should not be interpreted as causal and unraveling these mechanisms requires further study. For example, while we speculate about hormonal changes driven by oral contraceptives, we cannot determine which sex primarily governed the annual rhythm. Analogous to photoperiodic organisms like hamsters, where seasonal testes growth governs reproductive cycles (Elliott et al., 1972; Gorman and Zucker, 1995), a similar mechanism could potentially operate in humans, warranting rigorous research.

The potential influences of societal, technological, and environmental changes on birth rates are multifactorial and complex. Dissecting these influences to identify definitive causal relationships necessitates robust, multidisciplinary research. Given the global concern surrounding declining birth rates and their impacts on economies, healthcare systems, and societal structures (Goldstein et al., 2009; Bongaarts and Sobotka, 2012; Matysiak and Vignoli, 2013), this study contributes a new perspective. Consequently, addressing declining birth rates requires considering macro-level demographic and economic factors, as well as these nuanced influences. Understanding these dynamics may be essential for health services planning and policy development. For instance, consistent seasonal birth patterns could inform better resource allocation in maternity services. However, these patterns can shift over time, emphasizing the need for continuous monitoring and health services planning adaptability. Additional research is clearly necessary to fully comprehend the interplay of biological, societal, and environmental factors shaping human birth rates and their seasonal patterns and to inform whether associations drawn from such trends have biological meaning (Adsera, 2004; Mills et al., 2011).

## Supplemental Material

sj-docx-1-jbr-10.1177_07487304251384348 – Supplemental material for An Abrupt Mid-1970s Shift in UK Birth Seasonality and Its Implications for Chronobiological Studies Supplemental material, sj-docx-1-jbr-10.1177_07487304251384348 for An Abrupt Mid-1970s Shift in UK Birth Seasonality and Its Implications for Chronobiological Studies by Timothy J. Hearn and David Whitmore in Journal of Biological Rhythms

sj-xlsx-2-jbr-10.1177_07487304251384348 – Supplemental material for An Abrupt Mid-1970s Shift in UK Birth Seasonality and Its Implications for Chronobiological Studies Supplemental material, sj-xlsx-2-jbr-10.1177_07487304251384348 for An Abrupt Mid-1970s Shift in UK Birth Seasonality and Its Implications for Chronobiological Studies by Timothy J. Hearn and David Whitmore in Journal of Biological Rhythms
