# Supplementary material for: An Abrupt Mid-1970s Shift in UK Birth Seasonality and Its Implications for Chronobiological Studies
Source: J Biol Rhythms. 2025 Nov 26;41(1):28–41. doi: 10.1177/07487304251384348 (PMC12804422; doi:10.1177/07487304251384348)
Supplement: sj-docx-1-jbr-10.1177_07487304251384348 – Supplemental material for An Abrupt Mid-1970s Shift in UK Birth Seasonality and Its Implications for Chronobiological Studies [file sj-docx-1-jbr-10.1177_07487304251384348.docx]

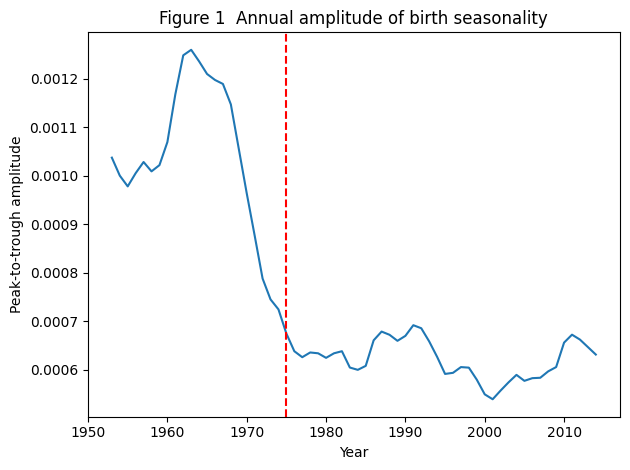


**Supplemental Figure 1. Annual amplitude of the birth-season component (1950 – 2014).**
Each point gives the peak-to-trough range, in births month⁻¹, of the STL-derived seasonal component for that calendar year. The vertical red dashed line marks 1975, the a-priori date of the demographic regime shift.


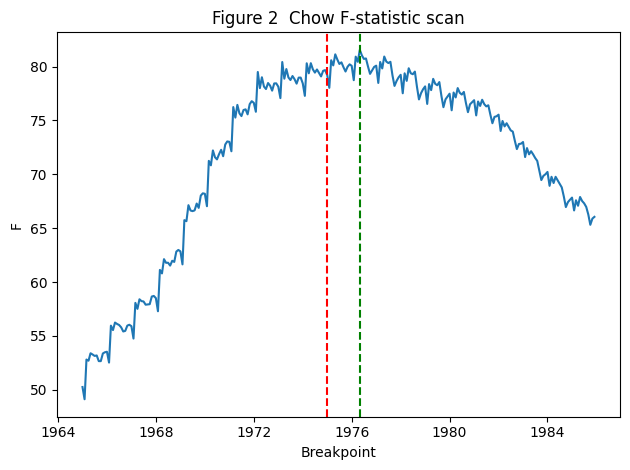


**Supplemental Figure 2. Chow F-statistic scan for a single structural break in the birth–environment regression.**
For every candidate breakpoint between January 1965 and December 1985 the Chow F-statistic compares the unrestricted regression on the full sample with two separate regressions split at that date. The horizontal axis is the assumed breakpoint; the vertical axis is $F(k,\text{ }n-2k)$ with $k=3$. The red dashed line marks 1975, while the green dashed line highlights the date that maximises $F$, denoting the most likely structural break under this test. Higher values indicate stronger evidence against parameter stability.

**
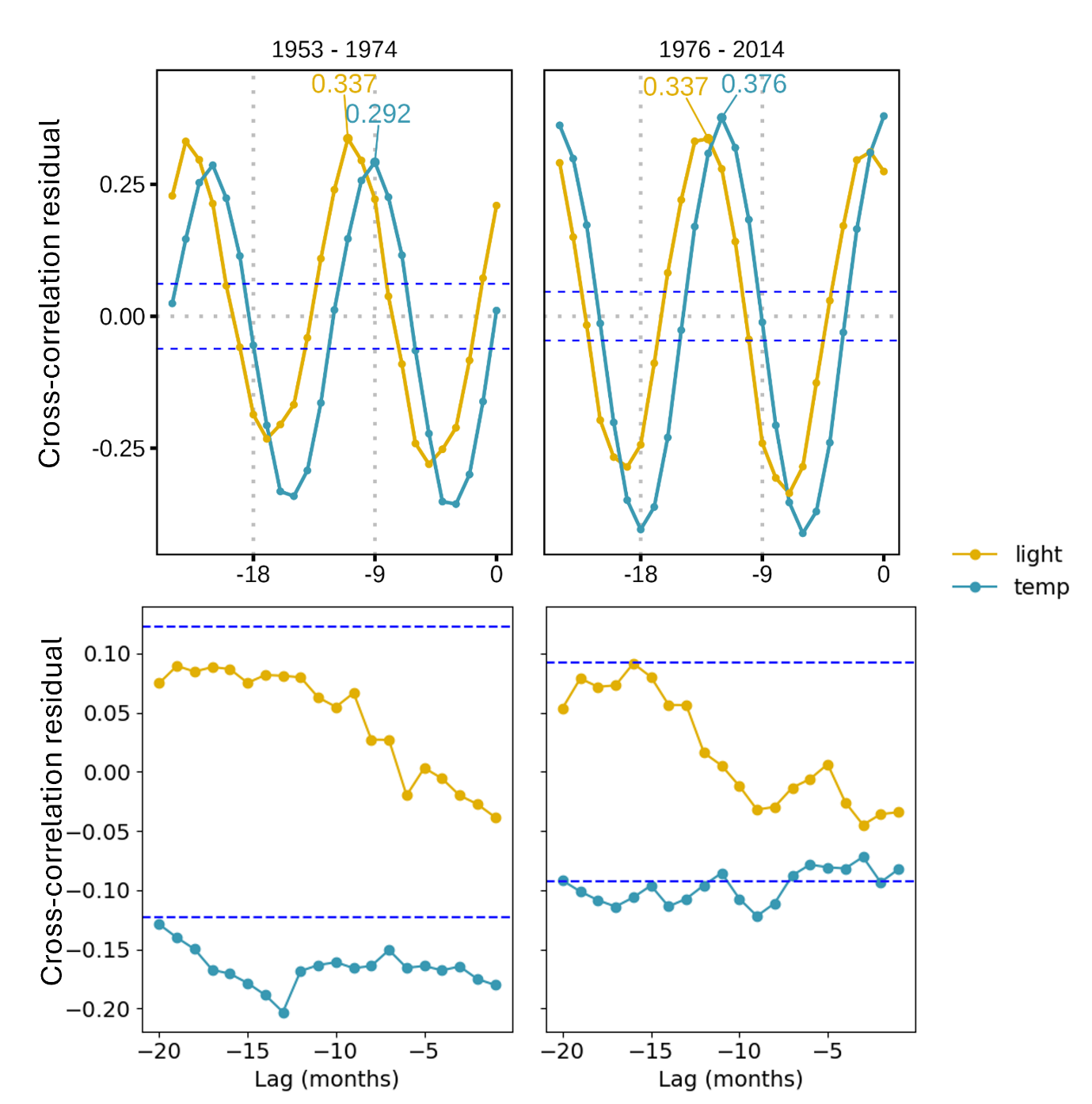
**

**B**

**A**

**Supplemental Figure 3. Cross‐correlation functions (CCFs) between UK birth rates and environmental drivers.**
**(A)** Raw‐series CCFs and **(B)** CCFs after removing the 12-month seasonal component by STL decomposition computed on z-scored monthly birth rates (BR) versus light (yellow) and temperature (blue) for two epochs (1953–1974, left; 1976–2014, right). Lags (x-axis) run from 0 to –24 months (months preceding BR), and dashed blue lines mark the significance cutoff ±2/√N (approximate 95 % bounds, N = number of months in each epoch). Filled circles denote the maximum CCF within the biologically plausible window.

**Supplemental Table 2. Top Chow candidates**

| **Test** | **Break Date (YYYY-MM-DD)** | **F-statistic** | **p-value** |
| --- | --- | --- | --- |
| **Chow (top candidates)** | 1976-05-01 | 81.56 | 1.11 × 10⁻¹⁶ |
|  | 1975-05-01 | 81.14 | 1.11 × 10⁻¹⁶ |
|  | 1976-06-01 | 81.04 | 1.11 × 10⁻¹⁶ |
|  | 1976-03-01 | 80.93 | 1.11 × 10⁻¹⁶ |

**Supplemental Table 3.** **Rolling-window peak-lag trends between environmental drivers and monthly birth rate.** For each 60-month window the lag that maximised $\mid r\mid\sqrt{n-2}$ (environment leading births by −1 to −20 months) was recorded and regressed on calendar time within the specified era. Negative slopes indicate that peak correlation lags moved earlier in the year, while positive slopes indicate a shift later over time. N windows refers to the number of 60-month windows contributing to the regression (i.e. number of peak-lag observations in the era)

| **Period (years)** | **Seasonal Adj.** | **Variable** | **Slope (months yr⁻¹)** | **StdErr** | **p-value** | **N windows** |
| --- | --- | --- | --- | --- | --- | --- |
| **1955 – 1974** | Raw | light | −0.0784 | 0.0364 | 0.0325 | 205 |
|  |  | photoperiod | −0.0097 | 0.0505 | 0.8473 | 205 |
|  |  | temperature | −0.0856 | 0.0473 | 0.0718 | 205 |
|  | Deseasoned | light | +0.1474 | 0.0788 | 0.0629 | 205 |
|  |  | photoperiod | +0.3502 | 0.0851 | 0.000056 | 205 |
|  |  | temperature | −0.2024 | 0.0895 | 0.0248 | 205 |
| **1976 – 2014** | Raw | light | +0.0804 | 0.0206 | 1.12 × 10⁻⁴ | 468 |
|  |  | photoperiod | +0.1709 | 0.0185 | 8.87 × 10⁻¹⁹ | 468 |
|  |  | temperature | +0.1922 | 0.0182 | 1.40 × 10⁻²³ | 468 |
|  | Deseasoned | light | −0.0604 | 0.0256 | 0.0188 | 468 |
|  |  | photoperiod | −0.0650 | 0.0265 | 0.0145 | 468 |
|  |  | temperature | −0.0055 | 0.0230 | 0.8110 | 468 |

**Supplemental methods**

**1.** **Seasonal adjustment.**

Because all four series exhibit strong annual seasonality, we obtained seasonally adjusted residuals using the Seasonal–Trend decomposition by Loess (STL) with a fixed 12-month period and robust weighting (Cleveland et al., 1990). For every variable *x* the seasonally adjusted value was

$$x_{t}^{\mathrm{ds}}=x_{t}-\hat{s}_{t},$$

Where $\hat{s}_{t}$ is the STL seasonal component. All subsequent analyses were carried out both on raw data and on these deseasonalised series. For each environmental series *x* and the birth-rate series *y* we slid a 60-month window across the record (January 1950 → December 2014).

**2. Annual amplitude of birth seasonality**

To visualise long-term changes in the strength of within-year birth timing, we calculated for every calendar year *y*

$$A_{y}=\max_{t\in y}\{s_{t}^{\text{BR}}\}-\min_{t\in y}\{s_{t}^{\text{BR}}\},$$

the peak-to-trough range of the seasonal component $s_{t}^{\text{BR}}$. The time-series $\{A_{y}\}$ was plotted with a reference line at 1975, the putative demographic regime shift.

**3. Chow breakpoint scan of static regression model.**

Using monthly data, the birth-season seasonal term was regressed on contemporaneous environmental seasonality:

$$\begin{matrix} & s_{t}^{\text{BR}}=\beta_{0}+\beta_{1}\text{ }s_{t}^{\text{photo}}+\beta_{2}\text{ }s_{t}^{\text{temp}}+\varepsilon_{t}, & & \end{matrix}$$

fitted by ordinary least squares (*statsmodels* 0.14). This was first estimated on the full 1955–2014 sample to obtain the unrestricted residual sum of square $\text{RSS}_{\text{full}} .$ To test whether the relationship experienced a discrete structural change, we performed a rolling Chow test (Chow, 1960). For candidate breakpoints we used a Monthly grid from January 1965 to December 1985, ensuring ≥ 5 years of data on both sides of any split. For each date $\tau$ the sample is partitioned into “pre” $(t\leq\tau)$ and “post” $(t>\tau)$. $\text{RSS}_{\text{split}}(\tau)$ is the sum of residual sums of squares from two separate OLS fits. With $k=3$ regressors (constant + two predictors) and total observations $n$,

$$F(\tau)=\frac{\left( \text{RSS}_{\text{full}} - \text{RSS}_{\text{split}} ( \tau) ) / k \right.}{\text{RSS}_{\text{split}}(\tau)/(n-2k)},$$

which follows $F_{k,\text{ }n-2k}$ under $H_{0}$ (parameter stability). *SciPy*’s F-distribution provided p-values. On the plot the a-priori breakpoint 1975 and the empirical maximum of $F(\tau)$ were highlighted.

**4.** **Residual Cross‐Correlation Analysis**
We assessed the temporal alignment of UK birth rates with two environmental drivers—photoperiod and temperature—using a two‐stage cross‐correlation approach. First, we computed the Pearson cross‐correlation function

$$\mathrm{CCF}(h)=\frac{\sum_{t=1}^{N-h} (X_{t}-X)(Y_{t+h}-Y)}{\sqrt{\sum_{t} (X_{t}-X)^{2}\text{ }\sum_{t} (Y_{t}-Y)^{2}}}$$

for lags $h=0,-1,\ldots,-20$ months on the *raw* z-scored series of monthly birth rate $(X)$

and each driver ($Y$) using Python’s NumPy routines. To guard against spurious peaks due to random noise, we overlaid a conservative significance cutoff at $\pm\frac{2}{\sqrt{N}},$ where N is the length of the epoch in months. Second, to remove the dominant annual cycle, we decomposed each series with STL (as described above) and subtracted the estimated seasonal component, yielding residuals $X_{t}^{'}$ and $Y_{t}^{'}$. We then recomputed $\mathrm{CCF}(h)$ for the same lag interval. From each raw‐series and residual‐series CCF curve we recorded the peak lag (the $h$ with maximal $\mid CCF\left( h \right)\mid$ ) and its correlation coefficient $r$. To quantify uncertainty in these peak‐$r$ values, we applied the Fisher $z$-transform

$$z=\frac{1}{2}\ln\text{ }\left( \frac{1+r}{1-r} \right), \mathrm{SE}_{z}=\frac{1}{\sqrt{N-3}},95\% \mathrm{CI}_{r}=\tanh(z\pm1.96\text{ }\mathrm{SE}_{z}).$$

**5. Rolling-window peak-lag trend analysis**

**Rolling-window peak-lag analysis.** Within a window we evaluated Pearson correlations at every negative lag $L\in[-20,-1]$. Because the number of overlapping observations $n(L)$ decreases with $\mid L\mid$, raw correlations are heteroscedastic. We therefore maximised

$$\text{score}(L)=\mid r(L)\mid\sqrt{\text{ }n(L)-2\text{ }},$$

the numerator of Student’s *t*, which is sufficient to rank lags yet cheaper to compute than the full statistic. Windows with $n\left( L \right)<3$ were skipped. The lag $L_{t}^{*}$ with the highest score became that window’s peak lag. Repeating the procedure for every window produced a monthly time-series $L_{t}^{*}$ for each environmental variable, separately for raw and deseasonalised data.

**Period segmentation.** Following the Chow test, we analysed two eras: 1955 – 1974 (prior to the mid-1970s structural break) and 1976 – 2014 (post-break modern era). Windows ending in the gap year 1975 were omitted to avoid contamination due to the breakpoint analysis.

For each era and variable we regressed the peak lag on calendar time:

$$L_{i}^{*}=\beta_{0}+\beta_{1}\text{ }t_{i}+\varepsilon_{i},t_{i}=\frac{\text{Date}_{i}-\text{Date}_{\text{start}}}{365.25}\text{ }[\text{years}],$$

using ordinary least squares (Python *statsmodels* v0.14). The slope $\beta_{1}$ (months · year⁻¹), its standard error, *t*-ratio and *p*-value were extracted. Slope direction indicates whether the environmental lead relative to births shortened ${(\beta}_{1}<0)$ or lengthened $(\beta_{1}>0)$ during the era.

**Supplemental references**

Cleveland RB et al. (1990) STL: A Seasonal-Trend Decomposition Procedure Based on Loess. *Journal of Official Statistics*. 6(1), p. 3-73.

Chow, G. C. (1960) “Tests of Equality Between Sets of Coefficients in Two Linear Regressions,” *Econometrica : journal of the Econometric Society*, 28(3), p. 591. doi: 10.2307/1910133.
